# Prevalence of hypertension and blood pressure profile amongst urban-dwelling adults in Nigeria: a comparative analysis based on recent guideline recommendations

**DOI:** 10.1186/s40885-019-0112-1

**Published:** 2019-04-15

**Authors:** Njideka U. Okubadejo, Obianuju B. Ozoh, Oluwadamilola O. Ojo, Ayesha O. Akinkugbe, Ifedayo A. Odeniyi, Oluseyi Adegoke, Babawale T. Bello, Osigwe P. Agabi

**Affiliations:** 1 Department of Medicine, Faculty of Clinical Sciences, College of Medicine, University of Lagos, Idi Araba, Lagos State, 100254 Nigeria; 2 Department of Medicine, Lagos University Teaching Hospital, Idi Araba, Lagos State, Nigeria

**Keywords:** Blood pressure, Hypertension, Prevalence, Nigeria

## Abstract

**Background:**

Hypertension is the major risk factor for cardiovascular diseases and prevalence rates are critical to understanding the burden and envisaging health service requirements and resource allocation. We aimed to provide an update of the current prevalence of hypertension and blood pressure profiles of adults in urban Nigeria.

**Methods:**

Cross sectional population-based survey in Lagos, Nigeria. Participants were selected using stratified multistage sampling. Relevant sections of the World Health Organization STEPwise approach to chronic disease risk factor surveillance were utilized for data collection. Blood pressures were categorized based on both the current American College of Cardiology/American Heart Association (ACC/AHA) 2017 guidelines and the pre-existing Joint National Committee on Hypertension 7 (JNC7) (2003) categories.

**Results:**

There were 5365 participants (51.8% female), age range of 16–92 years, and mean age ± SD 37.6 ± 13.1. The mean ± SD systolic and diastolic blood pressures were 126.8 ± 18.6 and 80.6 ± 13.2 respectively. There was significant correlation between both systolic and diastolic blood pressures and age (Pearson correlation 0.372 and 0.357 respectively and *p* = 0.000 in both instances). The prevalence of hypertension was 55.0% (3003) and 27.5% (1473) based on the ACC/AHA 2017 guideline and the JNC7 2003 guidelines respectively. Body mass index was positively correlated with systolic and diastolic BP (p = 0.000).

**Conclusions:**

Over half of the adult population in this major Nigerian city are classified to have hypertension by the recent guideline. There is an urgent need to develop and implement strategies for primordial prevention of hypertension (and obesity) and to restructure our healthcare delivery systems to adequately cater for the current and emerging hypertensive population.

## Background

Hypertension is the single most important risk factor for cardiovascular diseases (CVD) and a key driver of global disease burden. It is also a high-yield target to reverse the epidemic of non-communicable diseases (NCDs) globally. Major epidemiological studies exploring the antecedents to adverse non-communicable disease and cardiovascular outcomes consistently attribute the highest impact to hypertension. According to the Global Burden of Disease (GBD) study, in 2016, CVDs accounted for 5278.4 per 100,000 age-standardized disability-adjusted life years (DALYs). [1, 2] Hypertension-related diseases (specifically ischaemic heart disease and cerebrovascular disease) are the top two leading causes of DALYs and years of life lost (YLLs) globally. [1, 2]

A recent descriptive metanalysis based on the GBD 2015 study projected the prevalence rate of systolic blood pressure ≥ 140 mmHg as 20,526 per 100,000 (reflecting an absolute number of 874 million adults). [3] The annual death rate and loss of DALYs attributable to that BP level was 106.3 per 100,000 and 143 million respectively. [3] The estimated prevalence of hypertension in Nigeria from metanalysis of cross-sectional population and/or community-based studies published between 1980 and 2013, (using a cut-off definition of ≥140/90 mmHg) is 28.9% (30.6% in urban and 26.4% among rural dwellers). [4] The United Nations General Assembly (2011) reiterated prevention of risk factors as the foundation of the global response to NCDs, strongly advocating support and strengthening of national policies and healthcare systems. [5] Prevalence rates derived from direct enumeration of representative populations provide a credible evidence base for health services planning, allocation of scarce and competing resources, and economic derivations of disease burden. As the second decade since the most comprehensive national survey on hypertension concludes, it is imperative to understand the current blood pressure profile amongst adults in Nigeria. [6] Furthermore, with a current estimated population of 198 million and a projected increase to become the 3rd most populous country by 2050, with a population of 399 million, such data will highlight the absolute projected numbers of people with hypertension. [7] The present study was designed to update the profile of blood pressures in adults in urban Nigeria and determine the current prevalence of hypertension using the most recent recommended classification. [8]

## Methodos

### Ethical considerations

Approval of the study protocol was obtained from the Lagos University Teaching Hospital (LUTH) Health research Ethics Committee (HREC). Community entry protocol included notification of the Community Development Associations, permissions from community leaders, and creation of awareness in the study communities prior to study commencement. Notification of the intended study dates was disseminated to the community through the local government area representative about one week prior to the study commencement to sensitize the inhabitants and improve participation rates. All members of the community had equal opportunity to participate in the study. We obtained written informed consent from the head of household and from each individual participant.

### Study design, study site and sample size

This was a cross-sectional, population based survey conducted in the densely populated urban area of Lagos state, Nigeria. Lagos is the commercial capital of Nigeria with a population of about 14 million (approximately 10% of the national population). [9] Lagos has a population density of approximately 9300/km [2] (24,000/mile^2^) and is one of the fastest growing cities in the world. [10] Based on a previous metanalysis of the prevalence of hypertension in urban areas in Nigeria of 30.6%, we calculated a minimum sample size of 1287 participants with 99% level of confidence. [4] However, we aimed to exceed the largest sample size in any previous population-based study in Nigeria by recruiting a minimum of 5000 participants. [6, 11–18].

### Study participants and recruitment

We included adults aged 16 years and above residing in households within the community and excluded institutionalized populations such as those in prisons, hospitals, school dormitories and nursing homes. A stratified multistage random sampling approach was used to select eligible participants from selected households over an eight-month period (May to December 2017). We randomly selected eight densely populated mixed income areas from the 16 urban local government areas in Lagos State (Ikeja, Apapa, Mushin, Agege, Lagos Island, Lagos Mainland, Ifako-Ijaye and Oshodi-Isolo). Using the Enumeration Areas developed for the 1991 Population Census as the secondary sampling unit, four enumeration areas per local government areas were then randomly selected. With the aid of the official map from the National Population Commission for each enumeration area, we then randomly selected 200 households per enumeration area. Each selected household was the tertiary sampling unit from where consenting participants that met inclusion criteria were recruited.

### Data collection

Trained interviewers obtained relevant information from selected participants in their homes during a face to face encounter. The interviewers were experienced field workers and received study-specific training with respect to the protocol, questionnaire administration and standard measurements of blood pressure. The interviewers were supervised on the field by a public health physician with experience in large scale population surveys. Relevant sections of the World Health Organization STEPwise approach to chronic disease risk factor surveillance (WHO STEPS) were utilized to document the demographic data, body mass index (BMI), and as the protocol for blood pressure measurement in this study. [19] The questionnaire was piloted in a non-participating LGA and standardized prior to study commencement. Blood pressure was measured using Omron® sphygmomanometers calibrated before first use and at the beginning of every week thereafter. Blood pressure was recorded three times (one to three minutes apart) with documentation of the last two readings only. The average of the last two readings was utilized in this study.

### Data management and statistical analysis

Data was entered in Microsoft® Excel. All data was anonymized and coded prior to data analysis. Data checks for missing data were conducted and incomplete data discarded where sufficient data for the primary analyses was not available and attempts at re-collection (via telephone for historical data and in person for blood pressure re-measurement) was not feasible. In all, 213 (3.8%%) participant data were discarded. Blood pressures were categorized based on both the current American College of Cardiology/American Heart Association (ACC/AHA) 2017 guidelines and the pre-existing Joint National Committee on Hypertension 7 (JNC 7) categories. [8, 20] Of the 5365 participants included, weight and height data were available to compute the BMI in 5135 participants.

## Results

**Fig. 1:**
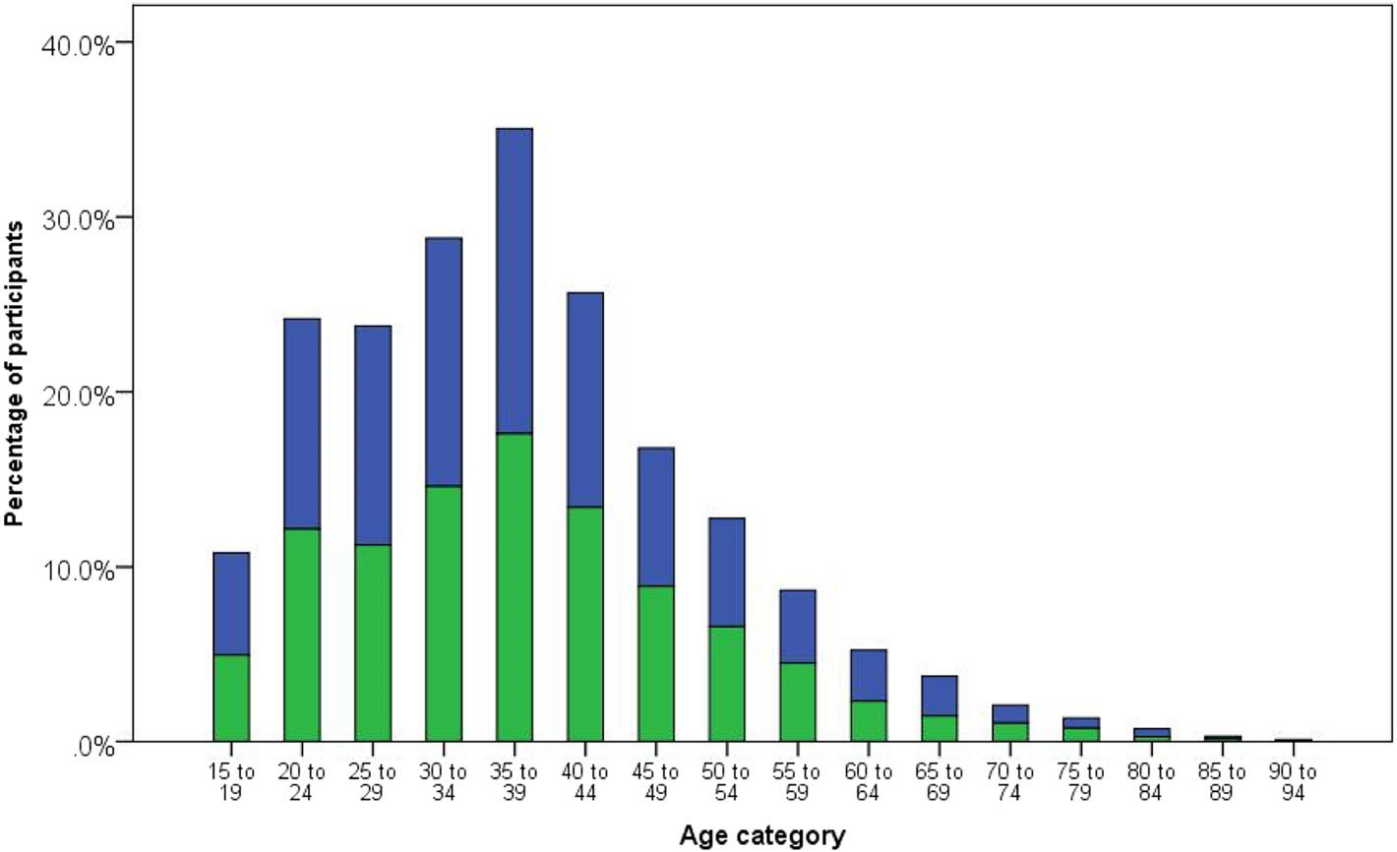
Distribution of study participants by age strata and gender. **Footnote:** Female (blue), male (green). Comparison of group differences in number of participants in age strata by gender distribution (Pearson Χ^2^ *p* = 0.36). 15–24 years: 939 (17.5%); 25–54 years: 3829 (71.4%); 55–64 years: 373 (7.0%); ≥ 65 years: 224 (4.2%)

The study included 5365 participants with an age range of 16–92 years, and comprised of 2781 females (51.8%) and 2584 males (48.2%). The age characteristics are shown in relation to gender in Table 1 and Fig. 1.

**Table 1:** Age characteristics and gender distribution of study participants

Characteristic	Total	Female	Male	*P* value
Number of participants, *(%)*	5365 (100)	2781 (51.8)	2584 (48.2)	
Age (mean ± SD), *years*	37.6 ± 13.1	37.5 ± 13.3	37.7 ± 12.9	0.61
Age range, *years*	16–92	16–92	16–90	
Age categories, *years*				
*Below 20*	291 (5.4)	163 (5.9)	128 (5.0)	
*20 to 39*	2999 (55.9)	1562 (56.2)	1437 (55.6)	
*40 to 59*	1710 (31.9)	848 (30.5)	862 (33.4)	
*≥ 60*	365 (6.8)	208 (7.5)	157 (6.1)	0.02

### Blood pressure profile

**Fig. 2:**
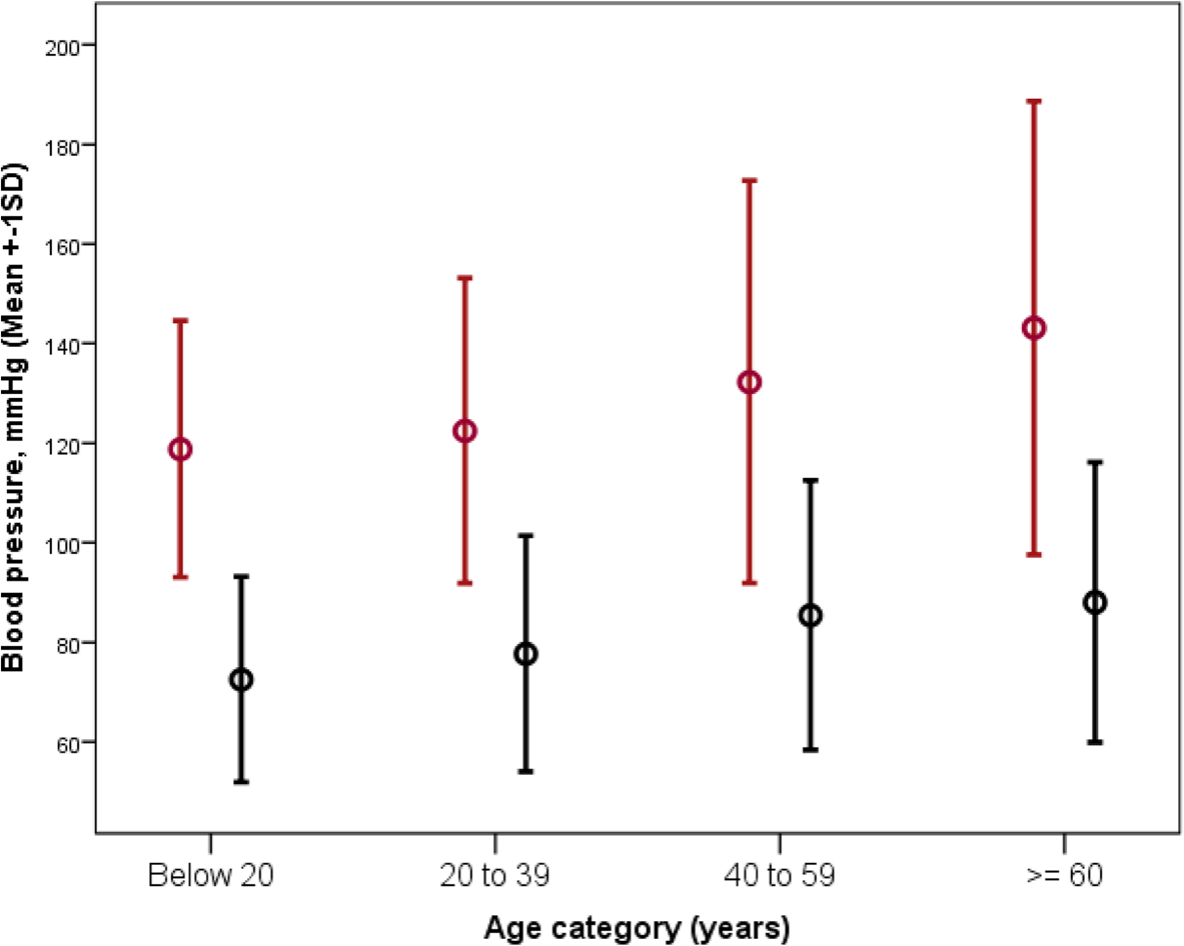
Comparison of mean systolic and diastolic blood pressures by age category. **Footnote:** Error bars represent systolic BP (red) and diastolic BP (black). Mean SBP in age categories: < 20 (118.8 ± 12.9), 20–39 (122.5 ± 15.3), 40–59 (132.3 ± 20.2) and ≥ 60 (143.1 ± 22.8). Mean DBP in age categories: < 20 (72.5 ± 10.3), 20–39 (77.7 ± 11.8), 40–59 (85.4 ± 13.5) and ≥ 60 (88.0 ± 14.1). Total numbers in each age category are shown in Table 1. ANOVA for between group comparisons; *p* = 0.000 for both SBP and DBP

**Fig. 3:**
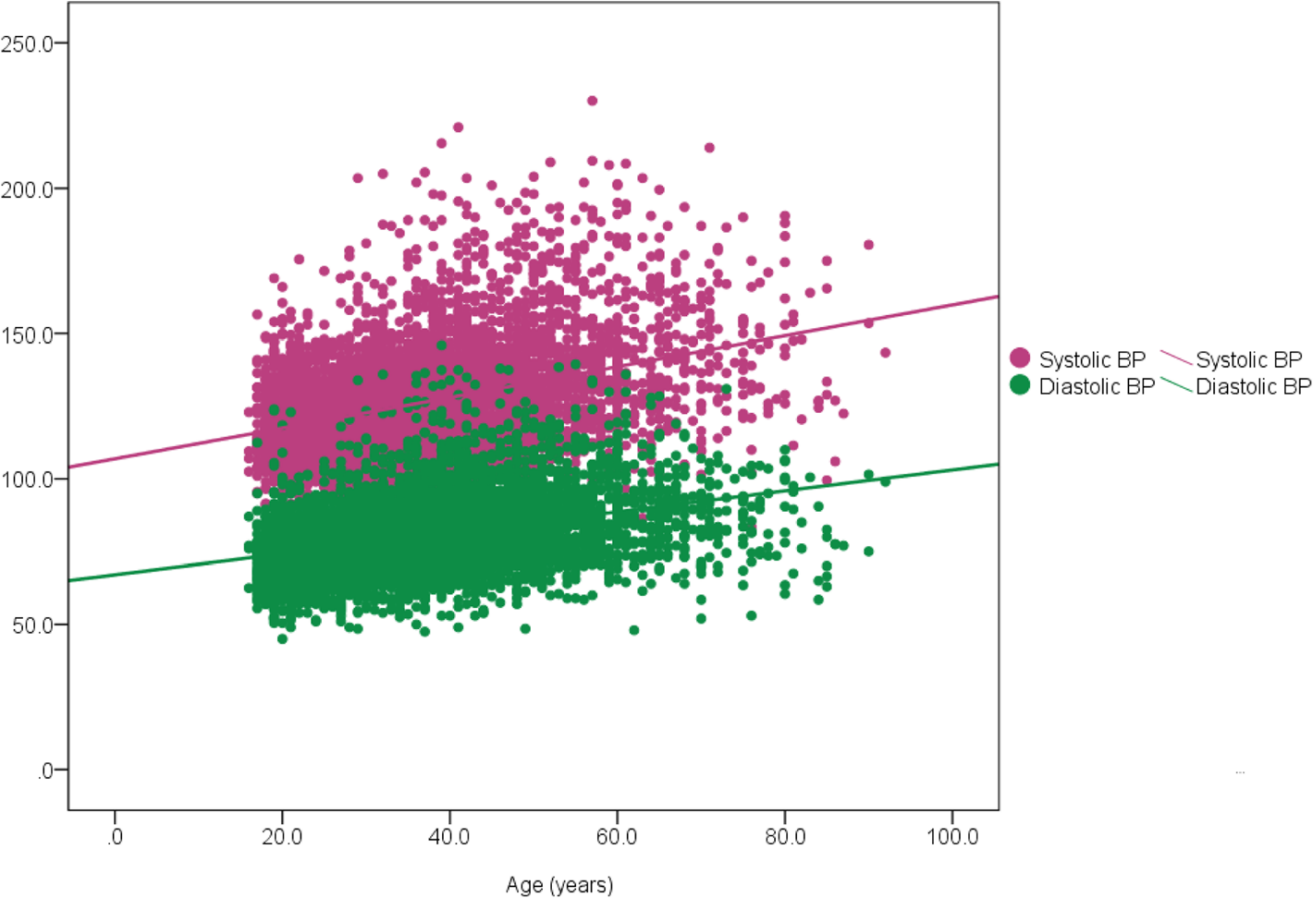
Association between systolic and diastolic blood pressures and age. Footnote: Scatterplot showing significant linear association between systolic BP (pink dots) and diastolic BP (green dots) and age (Pearson correlation; *p* = 0.000)

The mean (standard deviation) systolic and diastolic blood pressures of the study participants was 126.8 ± 18.6 and 80.6 ± 13.2 respectively. The blood pressure profiles including the range, median, distribution by major age categories, compared by gender is shown in Table 2 and Fig. 2. There was a significant correlation between both systolic and diastolic blood pressure and age (Pearson correlation 0.372 and 0.357 respectively and *p* = 0.000 in both instances) as shown in Fig. 3. The proportions of study participants within eight systolic BP categories (< 130, 130–134, 135–139, 140–144, 145–149, 150–154, 155–159, and ≥ 160 mmHg) is shown in Table 3.

**Table 2:** Blood pressure profile in study participants characterized by gender

Blood pressure	Total *N* = 5365	Female *N* = 2781	Male *N* = 2584	*P* value
Systolic blood pressure, *mmHg*				
*Range*	72.0–230.0	72.0–230.0	84.0–214.0	
*Mean ± SD*	126.8 ± 18.6	124.9 ± 19.6	128.9 ± 17.3	0.000^a^
*Standard error of the mean (SEM)*	0.26	0.37	0.34	
*Median*	124.0	121.5	126.5	
Diastolic blood pressure, *mmHg*				
*Range*	45.0–146.0	47.5–146.0	45.0–138.5	
*Mean ± SD*	80.6 ± 13,2	80.5 ± 13.4	80.7 ± 13.1	0.59
*Standard error of the mean (SEM)*	0.18	0.25	0.26	
*Median*	79.0	79.0	79.5	

^a^Significant difference (ANOVA; F = 62.1)

**Table 3:** Proportions of participants within systolic BP categories

Systolic BP, mmHg	Total (%) *N* = 5365	Female (%) *N* = 2781	Male (%) *N* = 2584
< 130	3351 (62.5)	1857 (66.8)	1494 (57.8)
130–134	570 (10.6)	249 (9.0)	321 (12.4)
135–139	378 (7.0)	166 (6.0)	212 (8.2)
140–144	339 (4.9)	137 (7.8)	202 (6.3)
145–149	185 (3.4)	87 (3.1)	98 (3.8)
150–154	134 (2.5)	67 (2.4)	67 (2.6)
155–159	86 (1.6)	41 (1.5)	45 (1.7)
≥ 160	322 (6.0)	177 (6.4)	145 (5.6)

### Prevalence of hypertension based on current and preceding guidelines

**Fig. 4:**
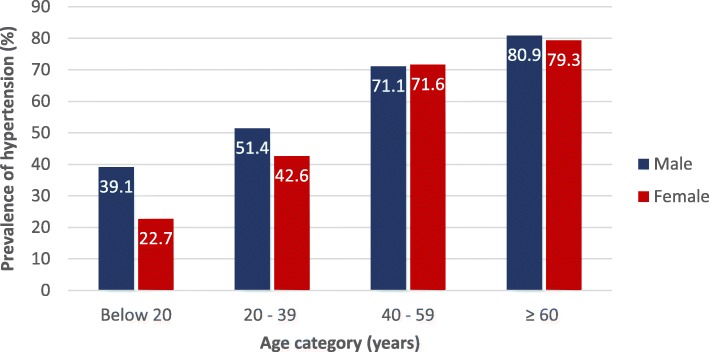
Prevalence of hypertension by age category and gender (ACC/AHA 2017 guidelines)

The prevalence of hypertension on the basis of the blood pressures measured at the time of the study is shown in Table 4. In order to account for the entire previously diagnosed hypertensive population whose BP may have been in control at the time of the measurement, previously known hypertensives whose BPs were normal (< 120/80) were counted as hypertensive. This did not significantly change the prevalence rates due to the relatively small numbers (see footnote at Table 4). The prevalence of hypertension (based on the current ACC/AHA 2017 guidelines) by age category and gender is shown in Fig. 4.

**Table 4:** Prevalence of hypertension using the ACC/AHA 2017 and the JNC7 (2003) guidelines

Category	ACC/AHA 2017	Statistics	JNC7 2003	Statistics
Overall (n = 5365)	3003 (56.0%)		1473 (27.5%)	
Females (n = 2781)	1475 (53.0%)	OR 1.28 (1.15–1.43); p = 0.000*	738 (26.5%)	OR 1.10 (0.98–1.24); *p* = 0.12
Males (n = 2584)	1528 (59.1%)		735 (28.4%)	
Age category, *years*				
*Below 20 (n = 291)*	87 (29.9%)		30 (10.3%)	
*20 to 39 (n = 2999)*	1404 (46.8%)		529 (17.6%)	
*40 to 59 (n = 1710)*	1220 (71.3%)	*p* = 0.000	699 (40.9%)	*p* = 0.000
*≥ 60 (n = 365)*	292 (80.0%)		215 (58.9%)	

Previously diagnosed (known hypertensives) whose BP was ‘normal’ at study evaluation and thus BP not categorized as hypertension: *n* = 56 using JNC7 and *n* = 26 by ACC/AHA 2017 (multiplied by 3.5% to accommodate 187 missing data = 58 and 27 respectively). Prevalence adjusted to include additional known hypertensives would be as follows: ACC/AHA = 3030/5365 i.e. 56.5% and 1531/5365 = 28.5%

The blood pressure categories based on the most recent ACC/AHA 2017 guidelines is compared to that based on the JNC 7 categories in Tables 4 and 5. [8, 20] The proportion of participants normotensive in the various age strata (for both JNC 7 and ACC/AHA 2017 i.e. BP < 120/< 80) are as follows: 15–24 (485/939 i.e. 51.7%), 25–54 (1178/3829 i.e. 30.8%), 55–64 (46/373 i.e. 12.3%), and ≥ 65 (17/224 i.e. 7.6%).

**Table 5:** Blood pressure categories based on ACC/AHA 2017 and JNC7 (2003) guidelines

ACC/AHA 2017 category	Frequency (*n*, %)	JNC7 2003 category	Frequency (*n*, %)
Normal	1726 (32.2%)	Normal	1726 (32.2%)
Elevated	636 (11.9%)	Prehypertension	2166 (40.4%)
Hypertension Stage 1	1530 (28.5%)	Stage 1 hypertension	930 (17.3%)
Hypertension Stage 2	1473 (27.5%)	Stage 2 Hypertension	543 (10.1%)

BP values for categories: Normal (for both guidelines) (< 120/< 80 mmHg); Elevated (120–129/< 80 mmHg), Stage 1 hypertension (130–139/80–89 mmHg), Stage 2 hypertension (> 140/90 mmHg) according to ACC/AHA 2017. Prehypertension (120–139/ or 80–89 mmHg), Stage 1 hypertension (140–159/ or 90–99 mmHg), Stage 2 hypertension (≥160/ or ≥ 100 mmHg) according to JNC7 2003

### Relationship of blood pressure and body mass index

BMI data are available for 5135 participants. The gender distribution for this subgroup of participants was similar to that of the group overall (female – 2671, 52%; male – 2464, 48%). The prevalence of hypertension (by JNC7 criteria – 1402/5135; 27.3% and ACC/AHA 2017 criteria – 2867/5135; 55.8%) was similar to that of the group overall (27.5 and 56% respectively, Table 4). There was a significant positive correlation between both systolic and diastolic blood pressures, and BMI (Pearson correlation 0.230 and 0,235 respectively, and *p* = 0.000 in both instances). Table 6 illustrates the significantly higher BMI in participants with hypertension based on both guidelines.

**Table 6:** Comparison of body mass index and prevalent hypertension based on ACC/AHA 2017 and JNC7 criteria

Participant category	ACC/AHA 2017			JNC7		
	Prevalence *n* (%)	HBP Yes BMI	HBP No BMI	Prevalence *n* (%)	HBP Yes BMI	HBP No BMI
All (*n* = 5135)	2867 (55.8%)	25.7 ± 5.6	23.3 ± 4.9	1402 (27.3%)	26.4 ± 5.9	24.0 ± 5.0
Female (*n* = 2671)	1411 (52.8%)	26.5 ± 6.0	23.5 ± 5.2	698 (26.1%)	27.3 ± 6.3	24.3 ± 5.4
Male (*n* = 2464)	1456 (59.1%)	25.0 ± 5.0	23.0 ± 4.6	704 (28.6%)	25.6 ± 5.5	23.6 ± 4.6

BMI was significantly higher for persons with hypertension using both criteria, and in both sexes (*p* = 0.000)

## Discussion

The impetus for the constantly moving target for defining hypertension is the resolve to reduce the negative impact that blood pressure elevation above ‘normal’ has on cardiovascular health. Given the strong evidence base for a consistent and independent association between higher blood pressure and the risk of stroke, heart failure, myocardial infarction, and chronic kidney disease, it is conceivable that the benchmark will continue to be redefined as research evidence accumulates in favour of lowering the threshold for initiating interventions for the primary prevention of adverse cardiovascular events.

The present study provides updated evidence of the high prevalence of hypertension in urban Nigeria (27.5% overall), corroborating the existing metanalytical data that approximately 1/3rd of urban dwelling adults in Nigeria and west Africa have hypertension. [4] In the 2014 report by Adeloye and Basquill, based on pooled analyses, the prevalence of hypertension (BP ≥ 140/90) was reported as 27.8% in sub Saharan Africa, 27.3% in west Africa (predominating in males as per this study). [4] The reported weighted mean SBP and DBP in their publication (129.6 and 78.0) is also very similar to our finding of 126.8 and 80.6 respectively. [4] Whereas the majority of studies included in their report were participants aged ≥20 years (mean 47.4 years), we included persons aged 15 years and above, with a mean age of 37.6, approximately one decade younger. The implication, considering the consistent linear association of age and both systolic and diastolic blood pressures (also demonstrated in this study), is the potentially higher prevalence of hypertension with advancing age, and the greater burden of major cardiovascular events overall. According to the JNC7 report, beginning at 115/75 mmHg, cardiovascular disease risk doubles for each 20/10 mmHg increment and lifetime risk of hypertension remains high even for those who are normotensive at 55 years. [20] As such, even the 10.6% of the participants in our study who are normotensive at 55 years still bear a 90% lifetime risk of becoming hypertensive. Put in the context of the additional projected population expansion that Nigeria will undergo on account of improved life expectancy and the increase in the elderly population proportion by 2030, we anticipate an even more enormous hypertension burden in the future. [7]

One of the objectives of this study was to determine the prevalence of hypertension based on the most current 2017 hypertension guidelines and the implications with respect to the difference in burden of hypertension requiring treatment in Nigeria. Compared to the JNC7 benchmark, using the ACC/AHA 2017 definition resulted in a doubling of the prevalence of hypertension (from 27.5 to 56.0%). The magnitude of the increase in prevalence was most profound in males (30.7% versus 26.5% in females, a difference of 30.7%), and in the age bracket 20–39 and 40–59 (differences of 29.2 and 30.4% respectively). Although the most affected demographics are fairly similar, there are differences when compared to data from the United States population as presented by Bundy and colleagues in their recent analyses. [21] Firstly, our study found a wider difference in prevalence between the 2017 and 2003 standards (28.5%) compared to theirs (13.4%, reflecting an increase from 32.0 to 45.4%). Furthermore, although males and the age bracket 40–59 were highlighted in both studies as being most markedly affected, we found that, in addition, the age stratum 20–39 in our urban population also had a 29.2% increase in prevalence, and females, those above 60 and even those below 20 all had a wider increase in prevalence (26.5, 21.1 and 19.6%) than the US figure of 13%. This probably represents the higher proportion of our population with BP in the 2003 prehypertension category and with diastolic blood pressures exceeding 80 mmHg and thus reclassified as having hypertension using the 2017 guidance.

On the basis of the current population of Nigeria being 190,632,261 (June 2017 estimate) [22], with 57.46% ≥ 15 years (109,537,297 persons), we project that approximately 61,340,886 have hypertension using the current diagnostic recommendations (30,122,757 based on JNC7), an additional burden of 31,218,129. (22) Adopting the latest ACC/AHA 2017 recommendations translate to an increase in the number of persons requiring antihypertensive treatment as a significant number of those with Stage 1 hypertension may bear a compelling reason to treat in addition to those with Stage 2 hypertension in whom treatment is presumably required. [8] The pros and cons of this new paradigm have been highlighted in several publications, pointing out the additional benefits of reduction in adverse cardiovascular events on one hand, but the increase in the economic burden and health manpower and infrastructure that is required to attend to the population concerned. [23–25] Despite the latter, the emerging high burden of adverse events such as stroke that have uncontrolled hypertension (either undiagnosed, untreated or poorly controlled) as the most important risk factor is sufficient reason to embrace the new direction. [26–28] Several in-depth analytical reports have suggested strategies to address this burden including adapting a total cardiovascular risk approach that targets both high and lower risk populations and developing less costly models of healthcare delivery (including universal health insurance coverage in the urban and rural setting) that can be rapidly implemented across the spectrum of healthcare settings (from primary to tertiary). [29–31].

Furthermore, our study aligns with existing data (including recent data from the Nigerian population) and reiterates the consistent positive correlation between BMI and blood pressure. [32] In this study, we demonstrated this association using both the JNC7 and ACC/AHA 2017 criteria. Despite criticisms of the utility of BMI in defining body fat distribution robustly with respect to the association with a risk of adverse cardiovascular events, the ease of deployment as a field tool is strength enough to promote its continuing applicability. Primordial prevention of obesity as a core public health initiative in our population is an important strategy if the contribution of adiposity to blood pressure profiles is to be curtailed.

### Limitations

We recognize that our sample population included urban black African dwellers in one Nigerian city, and that the data are thus largely representative of the scenario in an urban population. The advantage of the population selected is that Lagos is a multi-ethnic megacity with representation of the major ethnic groups, and social and lifestyle dynamics typical to urban populations across the world. The data are thus important in that it lends credence to the trend observed in other studies conducted in urban areas. Our study did not evaluate biochemical parameters (blood glucose and lipid profile) due to funding and logistic challenges, and we do acknowledge that providing this additional insight into the cardiovascular risk profile of our population could have improved the robustness of our data set. Treatment rates in previously diagnosed hypertension are also important to guide strategies to improve hypertension control in the population. We did not however obtain these data in the present study and understand that this gap in knowledge, while important, has not been addressed in this present study.

## Conclusions

Although the prevalence figures and the ensuing estimates of the absolute numbers with hypertension are alarming, put in context, the data are a tocsin and a call to action. There is an urgent need for stakeholders to develop, adopt or adapt and contextually implementable strategies for primordial prevention of hypertension on a population-wide scale in Nigeria, and to restructure our healthcare delivery systems to adequately cater for the current and emerging hypertensive population.

## Abbreviations

ACC: American College of Cardiology
AHA: American Heart Association
BMI: Body Mass Index
BP: Blood pressure
CVD: Cardiovascular disease
DALY: Disability–adjusted life years
GBD: Global Burden of Disease
HREC: Health Research Ethics Committee
JNC: Joint National Committee on Hypertension
LUTH: Lagos University Teaching Hospital
NCD: Non-communicable diseases
SD: Standard deviation
WHO: World Health Organization
YLL: Years of life lost
